# Gastrectasis. C—Treatment

**Published:** 1891-08-29

**Authors:** 


### THE PRACTITIONER'S MIRROR AND RETROSPECT.

[*The Editor will be glad to receive offers of co-operation and contributions from members of the profession. All letters should be addressed to* THE EDITOR, THE LODGE, PORCHESTER SQUARE, LONDON, W.]

### MEDICINE.

### GASTRECTASIS.

C.—TREATMENT.

In our two former articles we have considered the pathology and the diagnosis of dilatation of the stomach. The indications for treatment are sufficiently obvious, but it is by no means an easy matter to meet the difficulties with which we have to deal.

The first indication is to keep the stomach as empty as possible between meals, and to prevent the accumulation of semi-digested matter and mucus, and to endeavour to restore the normal peptic function. It is necessary that frequent lavage of the stomach be undertaken. If the secretion of hydrochloric acid be only diminished and not altogether absent, the contents will probably be free from fermentation, and the irritatingproducts of super-digestion, such as butyric acid and acetic acid will not be formed. Simply washing out of the stomach once daily before the principal meals of the day will suffice, the warm water being introduced and withdrawn several times until the water returning from the stomach is quite clear. Should there be much viscid mucus, it is desirable to add some carbonate of soda to the water used for lavage. If, from the absence or great diminution in the percentage of hydrochloric acid, which is the physiological antiseptic of the stomach, abnormal fermentation processes take place in the stomach, the characteristic odour of butyric and acetic acid will be recognised ; it then becomes necessary to add some suitable antiseptic to the water injected, such as $\beta$ naphthol, 1 in 1,000. Wolff recommends for this purpose either sodium salicylate or sodium borate.

Food should be given in small quantities at a time; it should consist of such easily digested liquids as are not prone to fermentation. Liquid food is more readily absorbed by the stomach, and is more easily passed on to the duodenum than are solid foods, which consequently are more liable to retention. But, as Dr. Fenwick says, it must not be forgotten that if the patient fails to fill the long-distended organ a sensation of faintness and craving will be induced that will tempt our patient to set at defiance all our directions. Consequently, we may be often obliged to give way to his solicitations, and allow food of a solid character. The main point is, however, to restrict the supply of food, and, in bad cases, this writer finds it a good plan to order nutrient enemata as well as small quantities of food by the mouth. Dr. Wolff states that milk diet, so freely and promiscuously ordered in all digestive troubles, is probably the most objectionable in this affection. Scraped meat on toast, white meats of fowl, stewed mutton, or boiled beef answer to excellent advantage when followed by fifteen to twenty drops of hydrochloric acid diluted in a wineglass of water. Toast in limited quantities is more readily digested than ordinary bread, while succulent vegetables are permissible. He gives as one of the principle articles of diet ordinary beef or mutton broth, to which, before being served, one or two raw eggs have been added and well beaten up ; and for stimulants, nothing but dry white wines or clarets are allowed at meals. Pastry, puddings, and sweets must be, of course, studiously avoided. This for the milder cases ; the graver cases, accompanied by almost complete apepsia, will be best maintained, by the mouth, by the above-mentioned broths, with eggs, followed by hydrochloric acid, while superadded to these he insists upon rectal alimentation.* Dujardin-Beaumetz recommends to avoid fish, game, shell-fish, crustaceans. The second indication is to endeavour to restore the muscular action of the stomach. Formerly, galvanism was employed for this purpose ; but of late, massage has been advocated. It is quite useless to order massage for a few days and expect any result. Abdominal massage should be practiced daily for several weeks, and then at longer intervals for some months. Unless one is prepared to persevere with it, it is better not to begin it at all. External faradization of the abdominal walls will bring about some improvement, but acting more or less indirectly on the stomach, the result is never very satisfactory, consequently Wolff has advocated internal faradization. The stomach electrode employed by him consists of a small nickel-plated brass olive screwed on to a flexible copper wire, about one yard in length, and insulated by being covered with rubber ; it is easily introduced, either by the patient himself, or by the medical attendant. Previous to its introduction the patient should drink a glass of water, and the current, which at the beginning should be feeble, should be gradually increased until peristaltic movements are experienced by the patient.*

* *Therap. Gaz.*, July, 1891.

That constipation, one of the most frequent and troublesome accompaniments of gastric dilatation, should require most careful treatment, is too obvious for further comment to be necessary.

And, finally, when a certain degree of improvement has been brought about, the patient must continue to exercise every precaution which will tend to promote easy and healthy digestion. The habit of careful mastication, and of giving plenty of time for his meals and between his meals, are of prime importance. It is well to administer some suitable intestinal antiseptic, such as the salicylate of bismuth, $\beta$ naphthol, or salol. Cod liver oil, fresh air, exercise, and tonics are very desirable.

